# Supplementary material for: DMRforPairs: identifying Differentially Methylated Regions between unique samples using array based methylation profiles
Source: BMC Bioinformatics. 2014 May 15;15:141. doi: 10.1186/1471-2105-15-141 (PMC4046028; doi:10.1186/1471-2105-15-141)
Supplement: Additional file 2 — DMRforPairs output for the comparison of A431-MCF7 and NA17018-NA17105. Please start from the HTML files in each folder. Available via the BMC Bioinformatics website. [file 1471-2105-15-141-S2.zip › 1394847754114233_MOESM2_ESM/NA17105M_NA17018F/figures/13789.pdf]

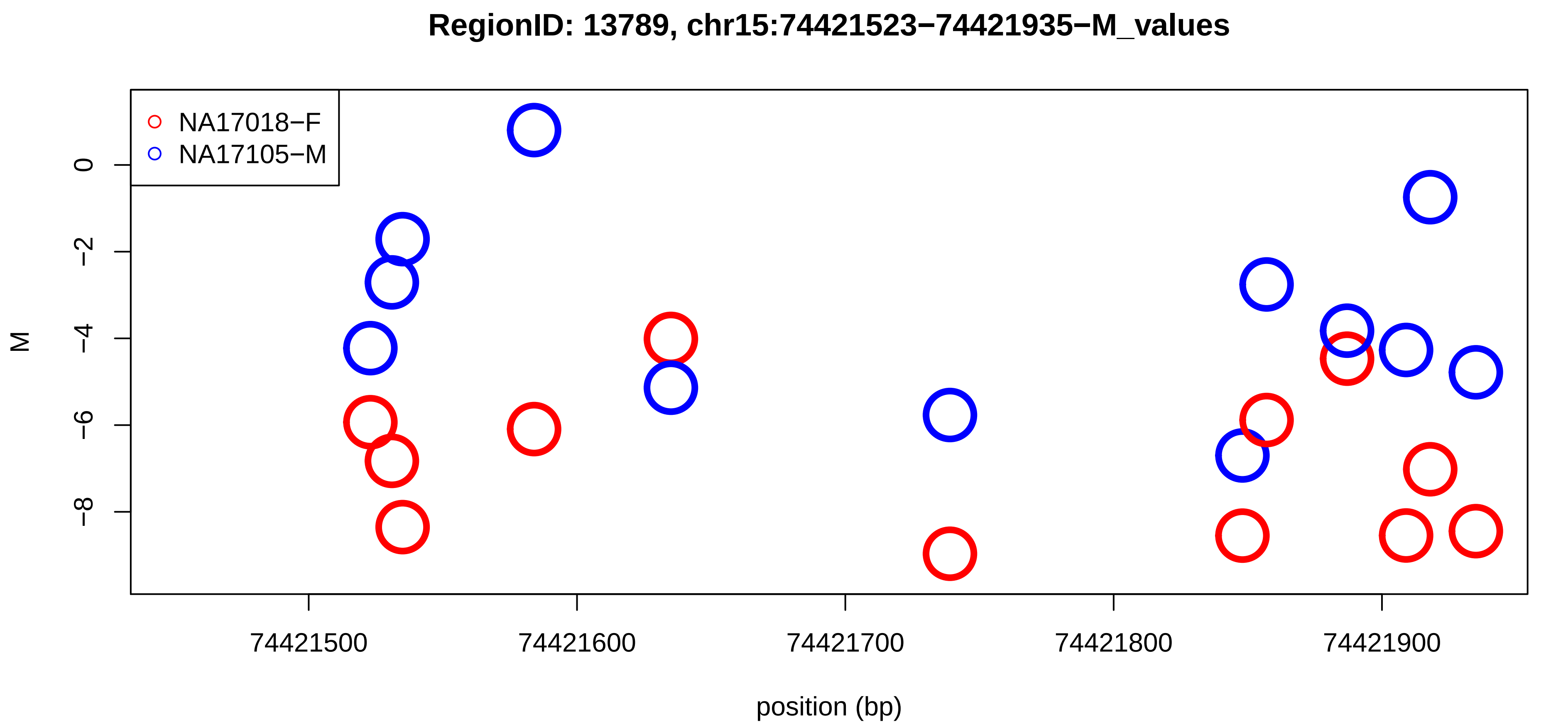
RegionID: 13789, chr15:74421523−74421935−M_values
NA17018−F
NA17105−M
M
0
−2
−4
−6
−8
74421500
74421600
74421700
74421800
74421900
position (bp)

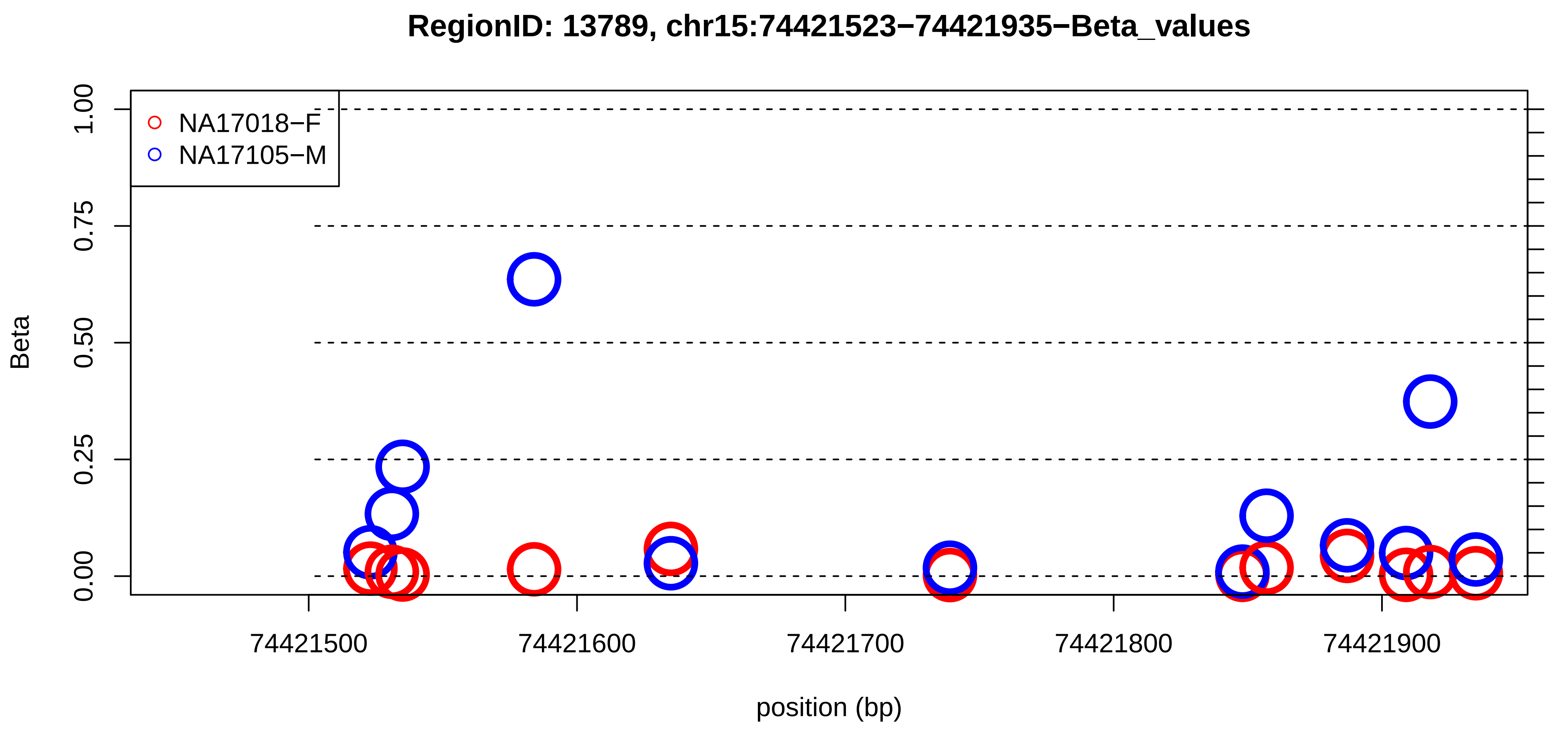
RegionID: 13789, chr15:74421523−74421935−Beta_values
NA17018−F
NA17105−M
Beta
1.00
0.75
0.50
0.25
0.00
74421500
74421600
74421700
74421800
74421900
position (bp)

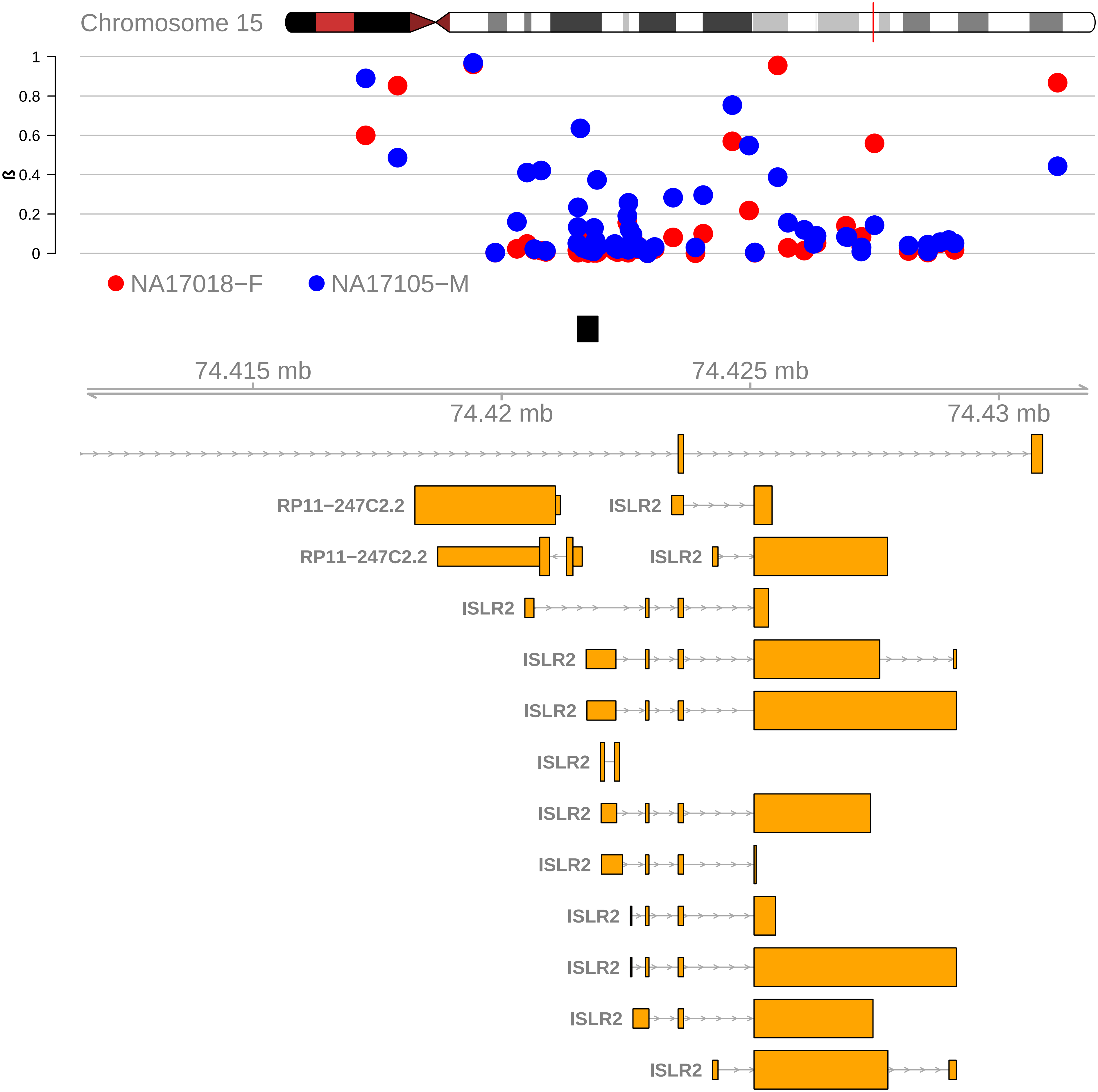

Chromosome 15
1
0.8
0.6
ß 0.4
0.2
0
NA17018−F
NA17105−M
74.415 mb
74.425 mb
74.42 mb
74.43 mb
RP11−247C2.2
ISLR2
RP11−247C2.2
ISLR2
ISLR2
ISLR2
ISLR2
ISLR2
ISLR2
ISLR2
ISLR2
ISLR2
ISLR2
ISLR2
